# The Nicotinic Acetylcholine Receptor as a Target for Antidepressant Drug Development

**DOI:** 10.1100/2012/104105

**Published:** 2012-04-24

**Authors:** Noah S. Philip, Linda L. Carpenter, Audrey R. Tyrka, Lawrence H. Price

**Affiliations:** Mood Disorders Research Program, Butler Hospital and Department of Psychiatry and Human Behavior, Alpert Medical School of Brown University, Providence, RI 02906, USA

## Abstract

An important new area of antidepressant drug development involves targeting the nicotinic acetylcholine receptor (nAChR). This receptor, which is distributed widely in regions of the brain associated with depression, is also implicated in other important processes that are relevant to depression, such as stress and inflammation. The two classes of drugs that target nAChRs can be broadly divided into mecamylamine- and cytisine-based compounds. These drugs probably exert their effects via antagonism at **α**4**β**2 nAChRs, and strong preclinical data support the antidepressant efficacy of both classes when used in conjunction with other primary antidepressants (e.g., monoamine reuptake inhibitors). Although clinical data remain limited, preliminary results in this area constitute a compelling argument for further evaluation of the nAChR as a target for future antidepressant drug development.

## 1. Introduction

Depression is one of the most common psychiatric illnesses in the world and has a significant public health impact. Despite the abundance of medications available, many affected patients do not achieve relief. In the largest comparative efficacy study of antidepressant treatment, the Sequenced Treatment of Alternatives to Relieve Depression (STAR*D) [1], about half of patients receiving initial treatment with antidepressants responded, but only a third reached remission, and up to a third never reached remission despite multiple trials of various medications and combinations. Most of the antidepressant drugs used in STAR*D directly increase synaptic availability of monoamines, often via reuptake inhibition. Clearly, new and better interventions that utilize novel mechanisms of action are needed.

Nicotinic acetylcholine receptor (nAChR) modulation is an area with significant promise for future antidepressant drug development. The nAChR literature builds upon studies from the 1970s that advanced a cholinergic theory of depression, in which excessive cholinergic signaling could lead to depressive symptoms. This area of research was largely abandoned due to issues regarding safety and tolerability of the compounds available at that time. More recently, with the development of new molecules targeting this system, significant interest in the antidepressant properties of nAChR modulation has reemerged. This paper reviews the available literature on the use of nAChR modulators for treating depression, building on previous reports [2]. Their use for other indications, such as smoking cessation and cognitive enhancement, has been addressed elsewhere [3–6].

## 2. The Nicotinic Acetylcholine Receptor

In their common ability to respond to acetylcholine binding, nAChRs are functionally related to muscarinic acetylcholine receptors (mAChRs). However, whereas mAChRs activate ion channels via G-protein coupling, nAChRs are ligand-gated ion channels and exhibit affinity for nicotine as well as acetylcholine. Activated nAChRs cause an influx of cations, which affect membrane polarity and intracellular messenger cascades [7]. nAChRs are distributed throughout both the peripheral and central nervous systems, although the focus here is on central nervous system (CNS) nAChRs. Formed of five pentameric units, nAChRs can be categorized as either high or low affinity. High-affinity nAChRs are heteromers of *α*- and *β*-subunits, are antagonized by compounds such as dihydro-*β*-erythroidine and mecamylamine and are stimulated by low doses of the *α*4*β*2 partial agonist varenicline. Low-affinity nAChRs, on the other hand, are *α* homopentamers and are antagonized by *α*-bungarotoxin and methyllycaconitine [7].

CNS nAChRs are widely distributed throughout brain regions associated with depression, such as the ventral tegmental area, locus coeruleus, and dorsal raphe nucleus. Their primary action is thought to be in the regulation of other neurotransmitter systems [8], particularly dopamine, via direct [9] and indirect effects [7, 10–13], and the n-methyl-D-aspartate (NMDA) glutamatergic system [14]. nAChRs are also involved in other neurobiological systems that are dysregulated in depression, such as the hypothalamic-pituitary-adrenal axis. nAChRs are found on presynaptic terminals of corticotropin-releasing factor (CRF) neurons [15], and nAChR antagonists can block CRF release [16]. Similarly, nAChRs appear to play a role in inflammation, which is the subject of increasing interest with regard to depression [17]. nAChRs regulate the so-called cholinergic ascending anti-inflammatory pathway, in which activation of the vagus nerve diminishes inflammation through decreased peripheral macrophage activity mediated via *α*7 nAChRs [18, 19].

## 3. nAChRs and Depression: Antidepressant Effects of Nicotine

Nicotine, the classic nAChR ligand, has been demonstrated to have antidepressant properties in both preclinical and clinical studies. In rodents, several studies of nicotine administration have identified antidepressant-like effects during the forced swim test (FST) and tail suspension test (TST) [20–23], two preclinical models used to investigate potential antidepressant effects of candidate compounds [24, 25]. Complementing these experiments, clinical studies have shown that transdermal nicotine can improve mood in both depressed smokers [26] and nonsmokers [27]. Antidepressant effects of nicotine are thought to be due to initial activation of the nAChR, followed by rapid desensitization that leads to long-term antagonism [28–32].

## 4. Targeting nAChRs for Depression

Most research targeting nAChRs for depression has focused on several parent compounds and their derivatives. The two principal compounds evaluated to date are mecamylamine, a nonspecific nAChR antagonist, and the plant alkaloid cytisine, which has been investigated for its effects on *α*4*β*2 nAChRs. The following sections are organized by parent compound, starting with a discussion of the preclinical literature supporting use for depression, then followed by clinical evidence, if available.

## 5. Mecamylamine

 Mecamylamine is a nonselective and noncompetitive antagonist at nAChRs. Derived from camphene, mecamylamine was first developed in the 1950s and utilized clinically as an antihypertensive agent [33], although its use was limited by anticholinergic side effects at therapeutic doses. After preliminary clinical studies that showed efficacy for mecamylamine in augmenting the efficacy of primary antidepressants [34], a number of preclinical studies evaluated its use in animal models of depression. Popik et al. [35] first demonstrated that mecamylamine, combined with the tricyclic antidepressant (TCA) imipramine or the selective serotonin reuptake inhibitor (SSRI) citalopram, improved immobility time during the FST. This study also investigated dihydrobetaerythiodine, a selective *α*4*β*2 nAChR antagonist, which increased the antidepressant-like effects of imipramine. Other studies consistently showed that racemic mecamylamine could improve performance on the FST or TST, either alone [36–39] or in combination with primary antidepressants [20, 37]. Administration of the s-enantiomer of mecamylamine, s-mecamylamine, also resulted in improvement in FST and behavioral despair tests [40]. These effects are driven by antagonism at the *α*4*β*2 nAChR, as knockout mice for this specific subtype of nAChR showed no effect of mecamylamine administration [37]. While most of these studies were positive, negative findings were reported by Andreasen and Redrobe [20]. They found no effect of mecamylamine when used alone or in combination with citalopram; only citalopram had significant effects on FST performance (Table 1).

 These preclinical studies laid the foundation for further clinical trials of mecamylamine as an augmenting agent for the treatment of depression. Shytle et al. [34] first showed that mecamylamine improved depressive symptoms and irritability in children and adolescents with Tourette's disorder, a neuropsychiatric condition characterized by multiple physical and vocal tics (i.e., stereotyped motor movements or vocalizations) [41]. George et al. [42] subsequently conducted an 8-week, double-blind, placebo-controlled trial of mecamylamine augmentation, administering mecamylamine 10 mg/day augmentation to patients with at least moderate depressive symptoms who had been on an SSRI for at least 3 months with little or no response. The investigators additionally hypothesized that smokers would have a more robust response to mecamylamine, as nAChRs are thought to be upregulated in smokers due to chronic nicotine self-administration [43]. Overall, mecamylamine augmentation resulted in significant improvement in depressive symptoms compared to placebo. In contrast to the investigators' hypothesis, there was a trend (*P* = 0.06) for nonsmoking patients to have a greater response than smokers, suggesting that mecamylamine might be more efficacious in the former group.

Larger commercial trials of racemic mecamylamine and s-mecamylamine as augmentation agents for depression were subsequently conducted. While the results are not in the public domain, outcomes from the racemic mecamylamine trial were reportedly positive, and phase II results for s-mecamylamine were also reportedly positive, with a large effect size [6]. However, preliminary reports from a large, multicenter, double-blind phase III trial failed to meet the primary endpoint of significant improvement in depressive symptoms [44]. There could be many reasons for this negative result, including issues differing efficacy in smokers versus nonsmokers. Cultural influences may also have impacted clinical trial outcome measures, as the phase II study was completed largely in India and, to a lesser extent, the United States, whereas the phase III study was conducted in European countries. Since this study is not in the public domain, it is difficult to evaluate possible explanations for the negative results, and it would seem premature to dismiss the potential antidepressant effects of mecamylamine without fuller access to the data.

## 6. Cytisine and Cytisine-Based Compounds

### 6.1. Cytisine

Cytisine, a partial agonist at the *α*4*β*2 nAChR [39], is extracted from the seeds of the *Cytisus labornum *(Golden Rain Acacia) [45]. It was first developed as an inexpensive smoking cessation agent in former Eastern Bloc countries in the 1950s and continues to be available as an over-the-counter smoking cessation agent in Russia. It constitutes the parent structure for other pyridine-like compounds, such as varenicline, dianicline, and sazetidine.

 Preclinical studies consistently have demonstrated antidepressant-like effects of cytisine in acute [39, 46] and chronic [39] rodent models. Cytisine administration has been linked to reduced expression of c-fos, an immediate early gene transcription factor reflecting neuronal activity [47] that downregulates in the context of chronic antidepressant treatment [48]. Other cytisine-based partial *α*4*β*2 nAChR agonists also have antidepressant-like effects [46], which further underscores the potential of this parent compound for treating depression. To our knowledge, there are no clinical studies investigating cytisine for depression, although the one available smoking cessation study found little effect of the drug on either smoking cessation or depressive symptoms [49]. These findings could be explained by cytisine's limited ability to affect CNS *α*4*β*2 nAChRs [50].

### 6.2. Varenicline

Varenicline, a partial agonist at the high-affinity *α*4*β*2 nAChR and full agonist at the *α*7 nAChR, was synthesized from its parent compound cytisine [51] in the pursuit of novel drugs for smoking cessation. In preclinical models, varenicline produced mild antidepressant-like effects during the FST, comparable to the SSRI sertraline but less than the TCA amitriptyline [52]. However, when administered in combination with sertraline, varenicline significantly improved FST performance. These effects were seen at lower doses of varenicline, which suggested an inverse dose-dependent effect [52]. One further study found no effect of varenicline on the FST and TST, but varenicline was administered in that study as a monotherapy, and most doses tested (0.01–1 mg/kg) were lower than those of other studies (0.178–5.6 mg/kg) [53] (Table 2). Large clinical trials for smoking cessation showed improvement in depressive symptoms associated with nicotine withdrawal [54, 55], and subsequent examinations found improved mood and cognition in subjects using varenicline for smoking cessation [56]. A small open-label study of varenicline augmentation in smokers with depression showed significant improvement in treatment-resistant patients [57].

One concern about the clinical use of varenicline has been neuropsychiatric side effects, such as increased depression or psychosis [60–62]. Although one report found that varenicline was associated with an increased rate of such events [63], other large studies have generally found no difference between varenicline and other medications for smoking cessation [64–67], findings that have been replicated in studies sponsored by the U.S. Food and Drug Administration [68]. An important consideration in evaluating this issue are the adverse effects upon mood of smoking cessation itself, which has been shown to induce major depressive episodes and suicidality in susceptible individuals [69].

### 6.3. Dianicline

Dianicline, also derived from cytisine, was developed to target high-affinity *α*4*β*2 nAChRs. The preclinical profile of dianicline is similar to that of varenicline, with its ability to affect dopamine release [70], although it has relatively weak CNS penetration [71]. To our knowledge, there are no studies evaluating the effect of this compound on preclinical models of depression. The one available clinical study of dianicline for smoking cessation was negative, with little effect on smoking outcomes or depressive symptoms [72], as might be expected for a medication with weak CNS penetration.

### 6.4. Sazetidine-A

Sazetidine-A is one of the newest compounds targeting the *α*4*β*2 nAChR. Sazetidine-A is thought to act in a different manner than other cytisine analogues, in that it binds to and desensitizes *α*4*β*2 nAChRs without activation and acts principally on the *β*2 subunit [59, 73]. Kozikowski et al. [58] showed that acute administration of sazetidine-A improved performance during the FST, to a degree comparable to that of the TCA desipramine. Several other recent studies have also shown antidepressant-like effects during the TST and FST [53, 59] (Table 2). These promising findings have prompted investigations into sazetidine-A analogues to evaluate potential antidepressant-like activity [74–76]. To our knowledge, there are no studies investigating sazetidine-A as an antidepressant augmenting agent in preclinical models, nor are there clinical studies investigating sazetidine-A for depression. However, given the preclinical data for related compounds, we anticipate that preclinical augmentation studies and clinical studies could yield positive results.

## 7. ***α***7 nAChR Agonists

 The structurally distinct *α*7 nAChR, composed of five identical *α*7 subunits, has been investigated for cognitive disorders and schizophrenia [33], but older data suggest that *α*7 agonists may have antidepressant activity. Studies from the late 1990s suggested that *α*7 receptors were required for the antidepressant effects of the SSRI fluoxetine [77], and it is possible that preclinical antidepressant-like effects of varenicline may be, in part, due to agonism at this receptor. Compounds targeting this nAChR have thus far generated contradictory findings. PNU-282987 has been shown to exhibit antidepressant-like effects when administered in combination with other antidepressants [78], but other studies have not replicated this [79]. While these studies may have been confounded by the specific mouse strains used [80], it is unlikely this compound will be tested in humans due to its potential for serious cardiac side effects [81]. Other *α*7 nAChR agonists that can improve FST performance, such as SSR180711, are still under investigation [79]. Although findings are still largely preliminary, they suggest that further study of the role of this receptor in depression is warranted.

## 8. Conclusion

There is clear evidence that nAChRs are a promising area for drug development in depression. Preclinical data document the greatest potential for compounds that target the *α*4*β*2 nAChR, with the sazetidine-A findings suggesting that the *β*2 component is necessary for antidepressant activity. All of these drugs appear to be most efficacious when administered in combination with other antidepressants and at low dosages. The relatively limited clinical data available to date indicate unlikely efficacy for cytisine and dianicline, possible efficacy for varenicline, and promising efficacy for mecamylamine as augmentation agents. It is unclear if these compounds will have differential efficacy in smoking or nonsmoking populations, and this should be an important consideration in future clinical trials. Taken together with the known limits of available antidepressant medications, the preclinical and clinical evidence provides a strong argument for future antidepressant drug development targeting this relatively unexplored receptor.

## Figures and Tables

**Table 1:** Evidence for MEC/S-MEC in mouse models of depression.

Study	Compound(s)	MEC/s-MEC dose	Results
Popik et al. [35]	MEC + CTP MEC + IMI	0.025 mg/kg	Improved FST

Rabenstein et al. [36]	MEC	1 mg/kg	Improved FST and TST

Caldarone et al. [37]	MEC	0.50, 0.75, or 1.0 mg/kg	Improved FST/TST
	MEC + AMI		Further improved FST/TST

Andreasen et al. [38]	MEC	3 or 10 mg/kg^a^	Improved FST/TST

Andreasen and Redrobe [20]	MEC + CTP	1, 3 mg/kg	No effect on FST/TST
	MEC + REB		

Mineur et al. [39]	MEC	1 mg/kg	Improved FST/TST

Lippiello et al. [40]	S-MEC	3 mg/kg	Improved FST

Notes: ^a^1–10 mg/kg doses tested, and only doses at 3 and 10 mg/kg with improved FST/TST.

Abbreviations: MEC: mecamylamine; CTP: citalopram; IMI: imipramine; AMI: amitriptyline: REB: reboxetine; S-MEC: s-mecamylamine; FST: forced swim test; TST: tail suspension test.

**Table 2:** Evidence for cytisine and cytisine-based compounds in mouse models of depression.

Study	Compound(s)	CYT-compound dose	Results
Mineur et al. [39]	CYT	1.5 mg/kg^a^	Improved FST/TST

Mineur et al. [46]	CYT	1, 1.5 mg/kg	Improved FST/TST
	3-pyr-CYT		Improved FST/TST
	5-Br-CYT		No effect on FST/TST^b^

Rollema et al. [52]	VNCL	0.178, 0.56, 1, 1.78, 3.2, 5.6 mg/kg	Improved FST
	VNCL + SERT	0.56, 5.6 mg/kg	Improved FST^c^

Kozikowski et al. [58]	SAZ-A	1 mg/kg	Improved FST

Turner et al. [53]	VNCL	0.01, 0.1, 1 mg/kg	No effect on FST
	SAZ-A	1 mg/kg	Improved FST^d^

Caldarone et al. [59]	VNCL	0.3, 1, and 3 mg/kg	Improved FST
	SAZ-A	1, 3, and 10 mg/kg	Improved FST

Notes:   ^a^0.2, 0.6, 1.0, and 1.5 mg/kg CYT tested.

^b^No CNS penetration.

^c^Low dose VNCL (0.56 mg/kg) combined with low dose SERT (1.78 mg/kg) produced no significant effect on FST performance.

^d^SAZ-A at doses of 0.05–0.5 mg/kg had no effect on FST performance.

Abbreviations: CYT: cytisine; 3-pyr-CYT: 3-[pyridin-3′-yl]-cytisine; 5-Br-CYT: 5-bromo-cytisine; VNCL: varenicline; SERT: sertraline; SAZ-A: sazetidine-A; FST: forced swim test; TST: tail suspension test.
